# The effect of isolation, fragmentation, and population bottlenecks on song structure of a Hawaiian honeycreeper

**DOI:** 10.1002/ece3.3820

**Published:** 2018-01-18

**Authors:** Joshua M. Pang‐Ching, Kristina L. Paxton, Eben H. Paxton, Adam A. Pack, Patrick J. Hart

**Affiliations:** ^1^ Tropical Conservation Biology and Environmental Science University of Hawaii at Hilo Hilo HI USA; ^2^ Department of Biology University of Hawaii at Hilo Hilo HI USA; ^3^ U.S. Geological Survey Pacific Island Ecosystems Research Center Hilo HI USA; ^4^ Department of Psychology University of Hawaii at Hilo Hilo HI USA

**Keywords:** avian malaria, birdsong, *Chlorodrepanis virens*, geographic variation, Hawai'i ‘amakihi

## Abstract

Little is known about how important social behaviors such as song vary within and among populations for any of the endemic Hawaiian honeycreepers. Habitat loss and non‐native diseases (e.g., avian malaria) have resulted in isolation and fragmentation of Hawaiian honeycreepers within primarily high elevation forests. In this study, we examined how isolation of Hawai'i ‘amakihi (*Chlorodrepanis virens*) populations within a fragmented landscape influences acoustic variability in song. In the last decade, small, isolated populations of disease tolerant ‘amakihi have been found within low elevation forests, allowing us to record ‘amakihi songs across a large elevational gradient (10–1800 m) that parallels disease susceptibility on Hawai'i island. To understand underlying differences among populations, we examined the role of geographic distance, elevation, and habitat structure on acoustic characteristics of ‘amakihi songs. We found that the acoustic characteristics of ‘amakihi songs and song‐type repertoires varied most strongly across an elevational gradient. Differences in ‘amakihi song types were primarily driven by less complex songs (e.g., fewer frequency changes, shorter songs) of individuals recorded at low elevation sites compared to mid and high elevation populations. The reduced complexity of ‘amakihi songs at low elevation sites is most likely shaped by the effects of habitat fragmentation and a disease‐driven population bottleneck associated with avian malaria, and maintained through isolation, localized song learning and sharing, and cultural drift. These results highlight how a non‐native disease through its influence on population demographics may have also indirectly played a role in shaping the acoustic characteristics of a species.

## 1 INTRODUCTION

In oscine Passerines (e.g., songbirds), song is a culturally transmitted trait that is acquired through social learning and imitation (Lynch, 1996). Thus, geographic variation in song can rapidly arise through the interplay of geographic isolation (Laiolo, 2008; Parker, Anderson, Jenkins, & Brunton, 2012; Robin, Katti, Purushotham, Sancheti, & Sinha, 2011; Valderrama, Molles, Waas, & Slabbekoorn, 2013) and localized generation of novel song elements or “memes” during song learning (Lynch, Plunkett, Baker, & Jenkins, 1989; Slabbekoorn & Smith, 2002). Natural (e.g., geographic distance, uninhabitable areas) or anthropogenic barriers (e.g., deforested landscapes, urban development) can resist or prevent meme flow between populations, much in the same way that barriers restrict gene flow (Lynch, 1996), and over time lead to the formation of unique dialects among populations across a landscape (MacDougall‐Shackleton & MacDougall‐Shackleton, 2001; Parker et al., 2012). Moreover, song divergence can be influenced by population size (Parker et al., 2012; Valderrama, Molles, & Waas, 2013) with smaller populations containing a lower diversity of memes for learning birds to sample from and an increased likelihood of losing novel song types (Lynch, 1996; Pavlova et al., 2012). Given the vital role of song as a signal between conspecifics for both territorial defense and mate attraction (Kroodsma & Byers, 1991), substantial geographic variation in a species’ song can give rise to behavioral barriers to gene flow (e.g., assortative mating) and ultimately lead to speciation (Grant & Grant, 1997; Wilkins, Seddon, & Safran, 2013).

Song divergence among populations can also arise from physical adaptations to environmental conditions, as a direct adaptation to the environment, or acoustic competition with other species. For example, ecological selection on bill size and shape to differing foraging niches can indirectly impose constraints on song features (e.g., frequency, trill rate) given the role of bill morphology in song production (Derryberry, 2009; Derryberry et al., 2012; Huber & Podos, 2006). In addition, birds may directly modify their songs, either temporally or spectrally, in response to competition for acoustic space with other species (e.g., Cardoso & Price, 2010) or the influence of habitat structures on the transmission of acoustic signals (e.g., Tobias et al., 2010). The acoustic adaptation hypothesis (Morton, 1975; Wiley & Richards, 1978) predicts that the natural attenuation and degradation of acoustic signals as they are transmitted through vegetation should lead to the selection of acoustic structures (e.g., pitch, frequency) that minimize interference and maximize transmission fidelity (Boncoraglio & Saino, 2007; Kirsche et al., 2011). Consistent with this prediction, birds in denser habitats have lower frequency, slower paced signals than birds in more open grassland habitats (reviewed in Boncoraglio & Saino, 2007). Over time, as conspecific populations occupy different habitats, signal structures may evolve to maximize transmission in each habitat, leading to acoustic variation across populations (Tobias et al., 2010).

In Hawai'i, little is known about how major social behaviors such as song vary within and among populations for any of the endemic Hawaiian honeycreepers (but see Sebastian and Hart 2017). Hawaiian honeycreepers are a group of songbirds (Subfamily Drepanidinea) recognized not only for their spectacular adaptive radiation (Lerner, Meyer, James, Hofreiter, & Fleischer, 2011), but also for their dramatic declines and high rates of extinction due to interacting pressures of habitat destruction and introduced diseases, predators, and competitors (van Riper & Scott, 2001). Landscape level habitat loss in Hawai'i has resulted in the isolation and fragmentation of remaining forest habitats for Hawaiian honeycreepers, and currently most Hawaiian honeycreepers are restricted to cool, high elevation forests where the prevalence of avian malaria (*Plasmodium relictum*) is limited due to low densities of mosquitoes that vector the non‐native disease (LaPointe, Goff, & Atkinson, 2010; van Riper, et al. 1987). However, in the last decade, a population of Hawai'i ‘amakihi (*Chlorodrepanis virens*) have shown tolerance to avian malaria, and as a result has been expanding in low elevation forests (Woodworth et al., 2005; Atkinson et al. 2013). Molecular genetic analysis of high and low elevation ‘amakihi populations indicates substantial genetic structuring, suggesting that low elevation ‘amakihi populations did not colonize from high elevation susceptible populations, but expanded from remnant low elevation populations (Eggert et al., 2008; Foster et al., 2007). In this study, we examined how isolation of Hawai'i ‘amakihi populations may have influenced acoustic variability in male song. We recorded and analyzed both qualitative song types and quantitative acoustic characteristics of ‘amakihi song from populations across an elevational gradient (10–1800 m asl) that parallels disease susceptibility. We hypothesized that isolation of ‘amakihi populations across landscapes of fragmented habitat and disease gradients would result in localized song sharing and divergence of songs among populations. We specifically addressed the following questions: (1) What is the degree of divergence among populations in both song‐type repertoires and acoustic characteristics of song? (2) What is the relationship between divergent song types among populations and the underlying physical acoustic characteristics of ‘amakihi song? (3) Which ecological features across the landscape are most strongly associated with differences in acoustic characteristics among ‘amakihi populations: (a) geographic distance between populations, (b) elevation of populations as a proxy for changes in disease susceptibility, or (c) habitat structure?

## 2 METHODS

### 2.1 Study species

**Figure 1:**
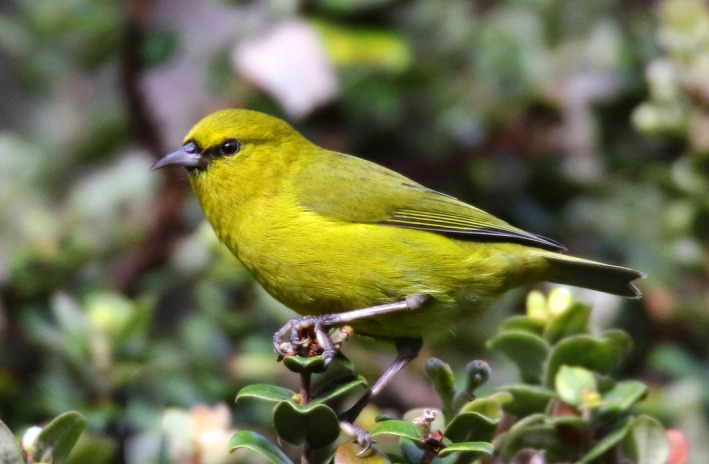
Photograph of a male Hawai'i ‘amakihi. Photograph credit: Ann M. Tanimoto

**Figure 2:**
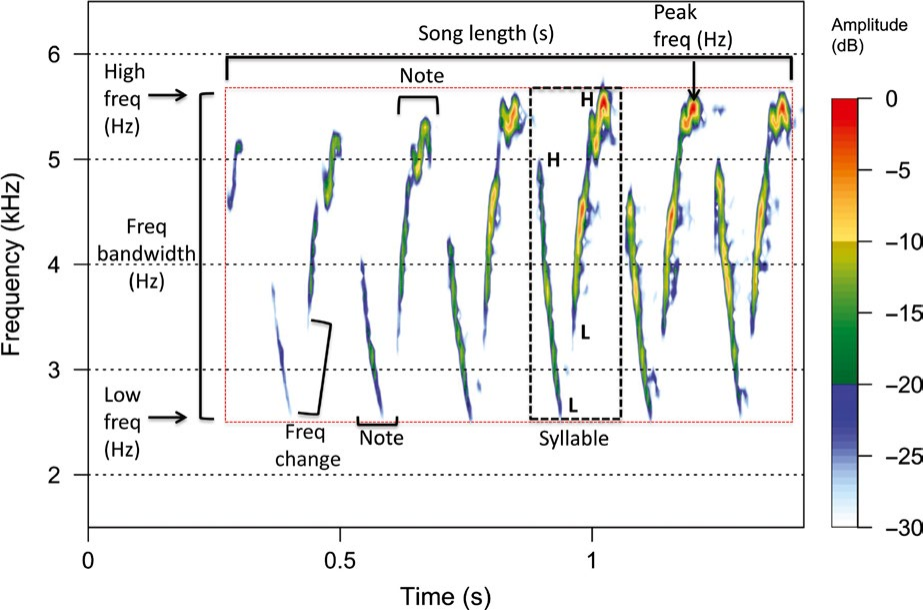
Representative Hawai'i ‘amakihi song spectrogram displaying the eight measured acoustic variables: song length (seconds), high frequency (Hz), low frequency (Hz), frequency bandwidth (Hz), peak frequency (Hz), total number of the repeated syllable (*n* = 6), number of notes per syllable (*n* = 2), and number of frequency changes per syllable (*n* = 2). Song type based on the repeated syllable within the song would be classified as “HLLH” with H and L reflecting a note starting or stopping at a high or low frequency, respectively

Hawai'i ‘amakihi (Figure 1) are common within native forest habitats on the islands of Hawai'i and Maui, and in low densities on Moloka'i (Lindsey, VanderWerf, Baker, & Baker, 1998). ‘Amakihi are a relatively sedentary, generalist honeycreeper species that feeds primarily on nectar and insects in vegetation ranging from the forest floor to the upper canopy (Lindsey et al., 1998). Males and females are sexually dimorphic with adult males having a bright yellow‐green plumage with black lores, while females are a drab olive color. We focused on the primary male song, a high‐pitched, undulating trill (Figure 2) that is used in both territory defense and courtship (van Riper, 1987).

### 2.2 Study sites

**Figure 3:**
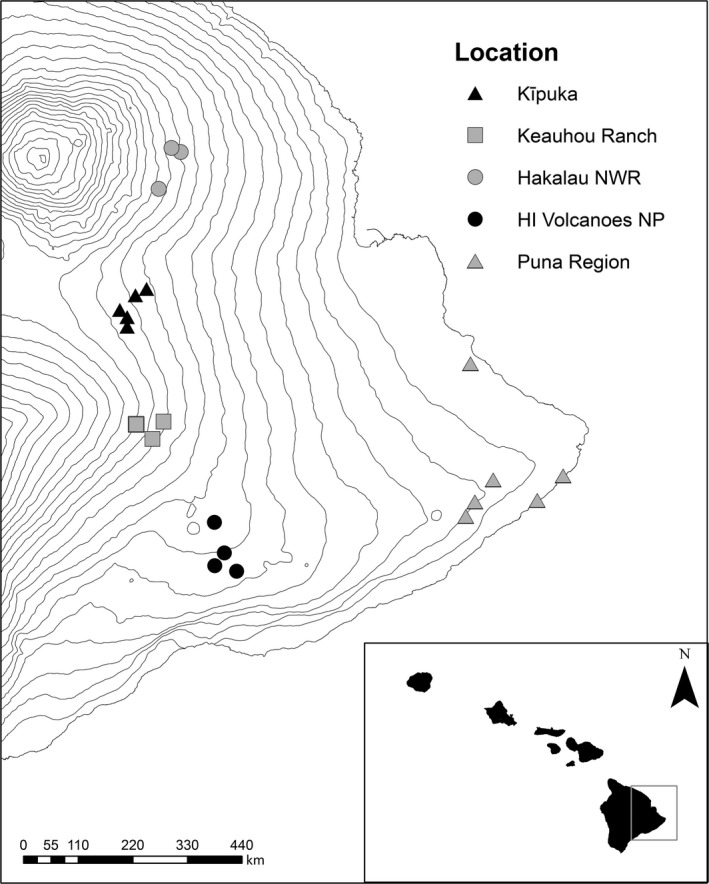
Map of the windward side of Hawai'i Island displaying five geographically distinct populations of ‘amakihi that vary across elevation (shown by contour lines). Within each population, male ‘amakihi song was recorded at 3–6 spatially separate recording sites

We recorded male ‘amakihi songs from five geographically distinct populations of ‘amakihi on Hawai'i Island that have little to no connectivity (Flasophler et al., 2010; Foster et al., 2007; Hart & Freed, 2003; Kilpatrick et al., 2006; van Riper, 1987). The five populations varied in elevation, including: (1) Puna district (PUNA) at 10–200 m elevation asl; (2) Hawai'i Volcanoes National Park (HAVO) at 800–1200 m elevation asl; (3) naturally fragmented forest areas known as kīpuka (KIPU) on the flanks of Mauna Loa between 800 and 1200 m asl; (4) Keauhou Ranch and adjacent Kīlauea Forests (KEAU) at 1500–1800 m asl; and (5) Hakalau Forest National Wildlife Refuge (HNWR) at 1500–1800 m asl (Figure 3). Within each population, we chose 3–6 spatially separate recording sites for subsampling. All sites were in humid wet forest (mean annual precipitation = 2,500–5,000 mm) dominated by ‘ōhi'a (*Metrosideros polymorpha*) and/or koa (*Acacia koa*) forest; however, there was some variability among sites in the assemblage of native and nonnative plants and the structure of understory habitat (e.g., open or closed understory habitats). While the overall distance between sampling sites within each population was fairly similar (approximately 4–13 km), the degree of habitat fragmentation varied among populations. Sites within KEAU and HNWR were within contiguous forest patches which contrasts with sites in HAVO and KIPU that consist of forest patches isolated from one another by recent lava flows that support scattered, small ‘ōhi'a trees and native shrubs. Sites in PUNA were of intermediate isolation from another, primarily separated by agriculture and urban development.

### 2.3 Acoustic recordings

We recorded songs from only adult males >2 years of age (identified by their bright yellow plumage and black lores) (Lindsey et al., 1998) between 0600 and 1200 hr from December 2011 to June 2013, corresponding to the ‘amakihi breeding season. During recordings, the microphone was positioned approximately ≤15 m from the singing male (depending on visibility and accessibility) to capture an entire bout of male song. Once the recording from an individual ‘amakihi was completed, the recorder moved >25 m away from the recorded male in order to avoid sampling the same male more than once. In two areas (i.e., HNWR and KIPU), we recorded songs from uniquely color‐banded individuals that were apart of ongoing demographic research (Knowlton et al., 2017, Paxton, unpublished data).

### 2.4 Recording equipment

‘Amakihi vocalizations were recorded using a Marantz PMD661 professional field recorder (Marantz America, LLC.) connected to a directional Sennheiser ME 67 shotgun microphone (Sennheiser Electronic Corporation) with a Rycote Softie cover mounted in a Rycote Pistol‐grip (Rycote Microphone Windshields Ltd) on a photographer's tripod. The Sennheiser ME 67 microphone has a frequency response of 40 Hz–20 kHz (±2.5 dB) and a maximum input sound level of 125 dB @ 1 kHz (THD = 1%). All tracks were recorded in 24‐bit WAV format at a 44.1 kHz sampling rate.

### 2.5 Song analyses

‘Amakihi songs were spectrographically imaged and analyzed using Raven Pro 1.4 software (Bioacoustics Research Program 2011). For each recording, we analyzed only those songs that were judged to be of high acoustic and visual quality (i.e., songs that were clear, contained minimal background noise, sound disturbance, static, or echoes, and did not overlap with other bird calls). All recordings were measured in Raven using a Hann window type with a DFT size of 600 samples, 91.1% time overlap, and a 3 dB Filter Bandwidth at 247 Hz.

To quantify and compare the acoustic characteristics of songs across all five populations, we measured eight standard parameters (e.g., Irwin, 2000; Robin et al., 2011): song length (total length of song from beginning to end, in seconds), high frequency (maximum, highest frequency level attained over the entire song, in Hz), low frequency (minimum, lowest frequency level attained over the entire song, in Hz), frequency bandwidth (frequency range, the difference between the high and low frequency, in Hz), peak frequency (dominant frequency, level at which the most energy is expelled within the song, in Hz), number of syllables, average number of notes per syllable, and average number of frequency direction changes per syllable (directional change, either ascending or descending, within a syllable) (Figure 2). Notes were defined as any continuous vocal utterance separated from other notes by >3 ms and a syllable as one or more notes grouped to form a single coherent unit (Marler & Isaac, 1961).

### 2.6 Identification of song types

We classified ‘amakihi songs into song types using an objective visually based song classification method based on a song's structure. Using an image file of the spectrogram of an individual ‘amakihi's song, we first identified the common syllable unit that was repeated within the ‘amakihi trill. We then identified the structure of the repeated syllable by mapping the direction of the frequency change(s) within each individual note using the letter “H” to designate the note in a high position, and the letter “L” to designate the note in a low position. For example, a repeated syllable with two notes, the first note descending (i.e., moving from a high position to a low position) and the second note ascending (i.e., moving from a low position to a high position), would be designated as HLLH (see Figure 2). We then grouped songs that had the same number of notes (e.g., 1, 2, 3) and structure (e.g., HLLH, HLHL), and visually compared songs within each group to determine whether the song types matched. This method allowed for a nonsubjective method to visually classify song types based on song structure.

### 2.7 Habitat quantification

We examined how habitat structure may influence acoustic characteristics of ‘amakihi song at five sites within two study locations, HNWR (*n* = 3 sites) and KEAU (*n* = 2 sites). We selected the two locations because they are both within continuous forest habitats with similar elevation, annual rainfall, and population densities, thus reducing confounding factors. Sites were classified as open or closed understories based on visual differences in the vertical structure of vegetation. Open understory forest habitats were dominated by 15‐ to 25‐year‐old plantation style (5 to 20 m spaced) koa trees with an understory of dense non‐native pasture grasses. In contrast, sites classified as closed understory forest habitats were comprised of old‐growth mixed ‘ōhi'a and koa trees with a primarily native understory. To verify our habitat classifications, we quantified differences in the vertical vegetation structure of habitats using a point intercept crown method (Bonham, 1989). We conducted a total of twenty‐four randomly placed 50‐m transects across the two study locations where song recordings were obtained. At every 2 m along the transect, a collapsible pole with 0.5 m marked intervals was used to record how many plant crowns intercepted the pole at eight categories from the ground: 0–0.5, 0.5–1, 1–2, 2–3, 3–4, 4–5, 5–10 m, and at >10 m.

### 2.8 Statistical analyses

To compare differences in song‐type repertoires among populations, we summed the number of individuals recorded singing each song type for each spatially separated site at each location (*n* = 21 sites). We used a Bray–Curtis measure of dissimilarity as a measure of distance between song types (Bray & Curtis, 1957). The Bray–Curtis coefficient measures the difference in abundance and richness of song types recorded between each pair of sites with a value of 1 indicating no shared song types and a value of 0 indicating the abundance and identity of all song types were the same between sites. We square‐root transformed the data to equalize the influence of common and rare song types in the dissimilarity matrix. We used three nonparametric multivariate techniques to assess differences in the composition of song types among populations using the R package vegan (Oksanen et al., 2017). First, to visualize differences in song types among populations, we used a nonmetric multidimensional scaling (NMDS) ordination plot with the metaMDS function. Based on stress levels of the ordination, we selected a two‐dimensional plot with the final ordination generated from 250 random starts. Second, we tested for differences in song‐type repertoire among populations using a permutational multivariate analysis of variance (PERMANOVA) with the adonis function. Last, we also compared differences in the variability of song‐type repertoire within populations using a permutational analysis of multivariate dispersion (PERMDISP) with the betadisper function. permanova tests for differences in the locations (e.g., centroids) of multivariate groups (Anderson & Walsh, 2013) while PERMDISP focuses on homogeneity of multivariate dispersions (e.g., distance of observations to their centroids) (Anderson, 2006). *p*‐values for test statistics (pseudo‐*F*) of the main effects test and a posterior pairwise comparisons were based on 9999 permutations.

To compare differences in the physical acoustic characteristics of songs among populations, we first assessed each acoustic variable for approximate normality and transformed, ln(*x*+0.1), two variables to remove skewness: number of syllables and average number of notes per syllable. We then averaged each acoustic variable to the level of site, and the relationship among populations was visualized using a principal component analysis (PCA) based on a correlation matrix. We retained principal component axes with eigenvalues >1 and calculated an acoustic distance matrix as the Euclidean distance between pairs of sites defined by the principal component scores. To test for differences in location and dispersion of acoustic characteristics of songs, we used a permanova and PERMDISP analysis, respectively.

To understand which physical acoustic characteristics are most strongly associated with different song types, we employed the nonparametric BIOENV procedure of Clarke and Ainsworth (1993) using the bioenv function in the R package vegan (Oksanen et al., 2017). BIOENV calculates a Spearman rank matrix correlation coefficient (*r*_*s*_) for each correlation between Euclidean distance matrices of all possible subsets of scaled acoustic variables and the Bray–Curtis dissimilarity matrix of song‐type repertoires. We determined the best combination of acoustic variables by the maximum *r*_*s*_ value, while we calculated significance of the BIOENV analysis using a Mantel test of the best subset of standardized acoustic variables and song‐type dissimilarity matrix. We then used a distance‐based redundancy analysis (dbRDA) in the R package vegan (Oksanen et al., 2017) to more formally model how similarities in song types among populations are structured based on the best subset of acoustic variables (McArdle & Anderson, 2001).

We examined the role of geographic distance, elevation as a proxy for disease susceptibility, and habitat structure on acoustic characteristics of ‘amakihi songs to understand underlying differences among populations. To determine the influence of geographic distance and elevation on acoustic characteristic, we used partial Mantel tests. An Euclidean distance matrix for both geographic distance and elevation was calculated between pair of sites, and then, each matrix was correlated with the acoustic distance matrix while controlling for the other factor. To determine the influence of vegetation structure on acoustic characteristics of ‘amakihi song, we conducted a PCA of the eight measured acoustic characteristics using only the individuals recorded at sites where detailed vegetation measurements were collected (*n* = 82 individuals). We then calculated an acoustic distance matrix as the Euclidean distance between pairs of individuals defined by the principal component scores with eigenvalues >1. We used a permanova to assess differences in acoustic characteristics between open and closed forest habitats. To verify differences in the vertical vegetation structure of sites classified as open and closed forest habitat, we calculated the proportion of vegetation “hits” at each height category across all transects within the same forest habitat classification. We used a chi‐squared test of independence to examine the association between forest habitat types and vertical vegetation structure.

All statistical analyses were conducted using the statistical program R version 3.3.2 (R Development Core Team 2016). Statistical significance was assumed at α = 0.05, and standard errors are provided unless otherwise noted.

## 3 RESULTS

We recorded songs from 348 individually identified male ‘amakihi across all five populations during the 2012 and 2013 breeding season, with an average of 69.9 ± 12.46 individuals from each population (Table 1). On average, we recorded 2.59 ± 0.12 songs per individual (range 1 to 23 songs). For the 214 individuals from whom two or more songs were recorded, we averaged all acoustic characteristics across songs. Based on an accumulation curve of song types within each population, sampling effort within each population appears to have captured the full range of song diversity, with the possible exception of Keauhou Ranch where the song curve does not reach an asymptote (Figure S1).

**Table 1:** Sample sizes for each location indicating the number of sites, individuals, and song types recorded. Song types are considered unique if they were recorded at only a single location. Also indicated is the proportion of individuals within a population singing a unique song type to that location, sharing a song type (unique or shared) with at least one other individual at the same location, or sharing a song type with at least one other individual at a different location

Location	No. sites	No. indivs	Total no. song types	No. unique song types	Proportion indivs unique song type	Proportion indivs share song type with indivs at same location	Proportion indivs share song type with indivs from different locations
Hawaii Volcanoes National Park	4	66	13	3	0.15	0.94	0.82
Hakalau National Wildlife Refuge	3	52	14	4	0.08	0.85	0.83
Keauhou Ranch	3	62	16	4	0.19	0.84	0.69
Kipuka	5	50	19	6	0.24	0.88	0.54
Puna Region	6	118	14	3	0.16	0.97	0.78

### 3.1 Analysis of qualitative song types

We recorded a total of 39 unique song types across all populations (Table 1). Fifty‐one percent of song types (*n* = 20) were recorded within only one population with on average 16% (± 3%) of individuals within a population singing a song type unique to their population. Most individuals (90% ± 3%) shared a song type with at least one other individual within their population ($\overset{¯}{x}$ = 7.35 ± 1.18 individuals within their population) (Table 1). Song types shared among populations (*n* = 19) were recorded on average within 2.95 ± 0.28 populations. Significantly fewer individuals (73% ± 5%) shared a song type with an individual(s) from a different population compared to shared song types within a population (Table 1) (paired *t*‐test; *t* = 3.14, *df *= 4, *p* = .03).

**Figure 4:**
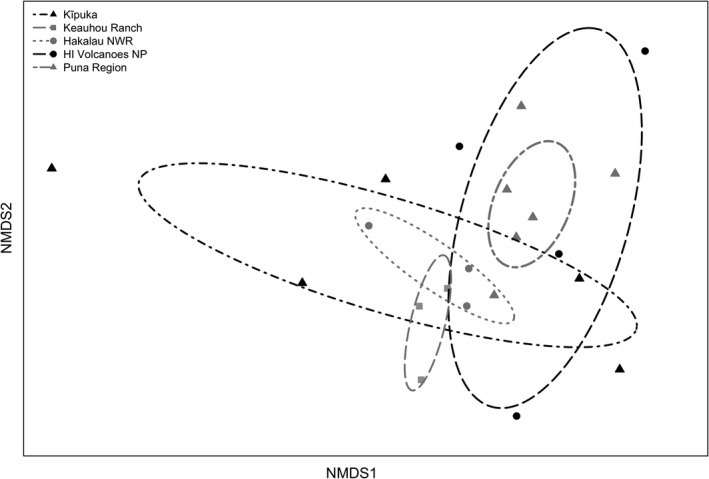
Nonmetric multidimensional scaling (NMDS) ordination of song types (stress = 0.13) based on Bray–Curtis square root transformed abundances of song types for 21 sites sampled across five populations. Each point represents a site, while the lined ellipses denote 95% confidence intervals for each population. Sites that are closer together on the ordination have greater similarity in the occurrence and frequency of song types than those further apart. The axes lack labels because an NMDS only shows information on the relative (rank‐order) similarities among points, and hence, the scale is arbitrary

**Figure 5:**
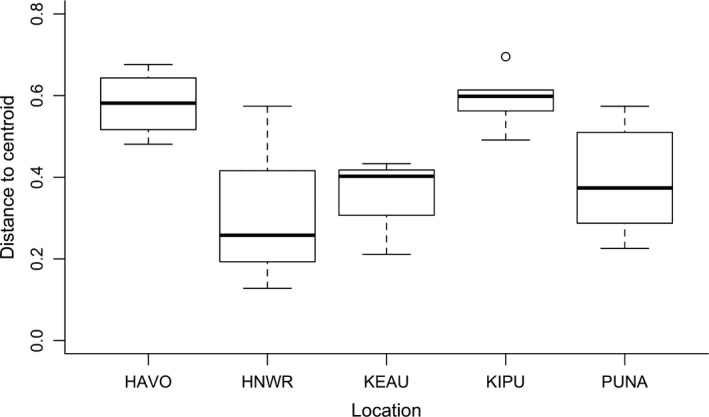
Song‐type variability (multivariate distance‐to‐centroid) is greatest within Hawai'i Volcanoes National Park (HAVO) and the kīpuka region (KIPU), both representing naturally fragmented landscapes compared to contiguous forest patches within Hakalau National Wildlife Refuge (HNWR) and Keauhou Ranch (KEAU). Sites in the Puna region (PUNA) have intermediate isolation from one another, primarily separated by agriculture and urban development. Post hoc pairwise comparisons of 9999 permutations found that variability within KIPU was significantly greater than all other locations, except HAVO. In addition, variability within HAVO was significantly greater than all other locations, except KIPU and HNWR. All other locations had similar variability

Nonmetric multidimensional scaling ordination revealed differentiation in song‐type repertoires among some populations, but with considerable overlap in song repertoires for many populations (Figure 4). permanova supported the ordination, indicating significant differences in song repertoires among populations (*F*_4,16_ = 1.58, *p* = .01, *R* ^2^ = .28). Pairwise comparisons showed that differences among populations were driven by significant differences between song types recorded at low elevation sites in the Puna Region compared to mid and high elevation sites at Hakalau National Wildlife Refuge (*p* = .03), Keauhou Ranch (*p* = .01), and the kīpuka region (*p* = .01) (Table S1). The greatest variability in song‐type repertoires within‐populations (e.g., multivariate distance‐to‐centroid) was found in those populations that occur in naturally fragmented landscapes, Hawai'i Volcanoes National Park and the kīpuka region (PERMDISP; *F*_4,16_ = 3.91, *p* = .02) (Figure 5) (Table S1).

### 3.2 Analysis of quantitative acoustic characteristics

**Figure 6:**
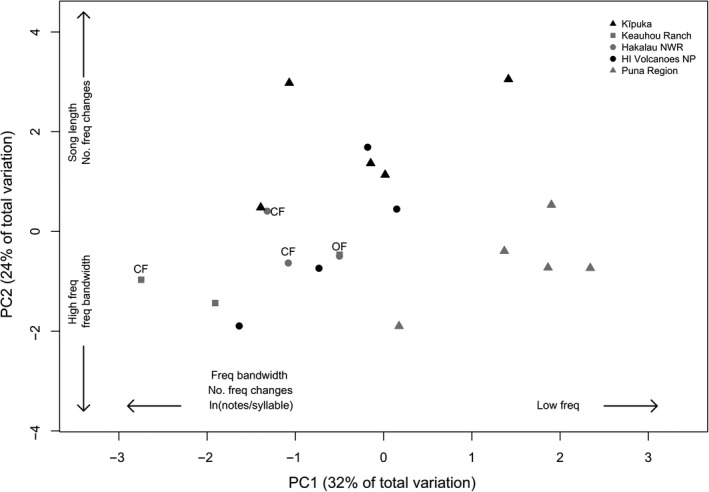
PCA ordination of the standardized acoustic variables averaged for each site within a population. Fifty‐six percent of the variation in acoustic characteristics of Hawai'i ‘amakihi songs is explained by the first two principal component axes. Acoustic characteristics with the greatest PCA loadings (>0.35) for each principal component axis are displayed on figure (see Table 2 for PCA loadings). Individuals within the Puna region differed from other populations by less complex songs (e.g., decreasing number of frequency changes, notes per syllable, and song length) and the frequency of songs (e.g., higher high and low frequency). Sites within Keauhou Ranch and Hakalau National Wildlife Refuge are also classified as open (OF) or closed (CF) forests based on the vertical structure of habitat at the site

We found a high degree of variability in the acoustic characteristics of Hawai'i ‘amakihi songs among populations. Songs ranged from simple songs with no undulation, measured as the average number of frequency changes within each repeated syllable, to complex songs containing up to 6 frequency changes per syllable ($\overset{¯}{x}$ = 2.56 ± 0.07). Song length also varied with the number of times a syllable was repeated within a trill ranging from only five repeats to longer songs comprised of a syllable repeated up to 18 times ($\overset{¯}{x}$ = 9.13 ± 0.11). A PCA analysis of the eight measured acoustic variables showed that four principal component axes explained 83% of the variation in acoustic characteristics (Table 2). Based on an acoustic distance matrix of the four principal component axes, we found significant differences in acoustic characteristics of ‘amakihi songs among populations (Figure 6) (permanova; *F*_4,16_ = 3.80, *p* = .0001, *R* ^2^ = .49). Similar to pairwise comparisons of song types, differences in acoustic variables among populations were driven by differences between individuals recorded at low elevation sites in the Puna region compared to all other populations (Table S1). However, the variability of acoustic characteristics within a population was the same among populations (PERDIST; *F*_4,16_ = 0.56, *p* = .69).

**Table 2:** Principal component loading values of eight acoustic variables measured from Hawai'i ‘amakihi songs recorded across five populations. Four principal component axes had eigenvalues >1 and explained 83% of the variation in acoustic variables. Acoustic characteristics with the strongest PCA loadings (>0.35) are in bold

Acoustic variables	PC1	PC2	PC3	PC4
Song length	−0.31	**0.44**	0.20	**0.47**
Low freq	**0.52**		**−0.44**	0.14
High freq	−0.15	**−0.62**	−0.30	
Freq bandwidth	**−0.51**	**−0.36**	0.16	
Peak freq	0.34	0.20	**0.42**	**−0.48**
Freq change	−0.33	**0.37**	−0.35	0.17
ln(no. syllables)		−0.32	**0.59**	**0.38**
ln(notes per syllable)	**−0.35**	0.12		**−0.59**
Eigenvalue	2.54	1.94	1.16	1.01
Proportion of variation	0.32	0.24	0.15	0.13
Cumulative proportion	0.32	0.56	0.71	0.83

### 3.3 Relationship between song types and acoustic characteristics

**Figure 7:**
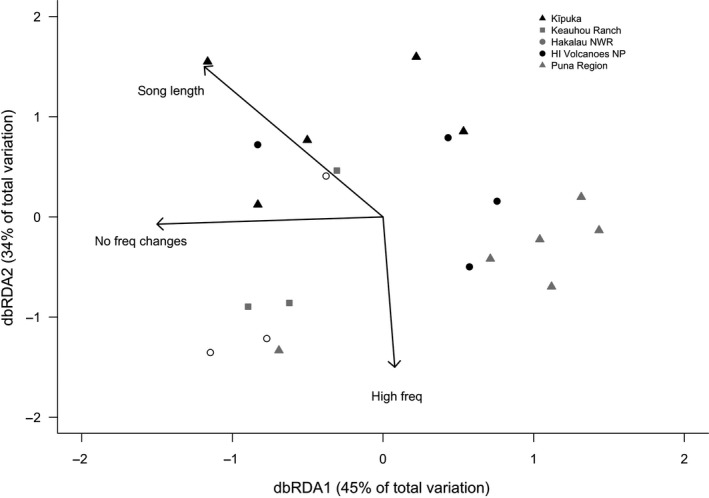
Distance‐based redundancy analysis (dbRDA) ordination of song‐type dissimilarities overlaid with acoustic characteristics that best predict the variation in song types. The number of frequency changes in a song type and the highest frequency obtained within a song type decrease along the *x*‐axis and *y*‐axis, respectively, while song length is associated with both axes. Most individuals within low elevation Puna region sites have song types that are differentiated from other higher elevation populations, except Hawai'i Volcanoes National Park, based on differences in song length and the number of frequency changes in a song, two measures of song complexity

The BIOENV analysis identified three acoustic characteristics that best predicted the distances among population song‐type repertoires (Figure 7) (partial mantel test; *p* = .001, *r*_*s*_ = 0.44). Song length and the number of frequency changes within a syllable, both measures of song complexity, along with the highest frequency obtained within a song explained 44% of the variation in the song‐type dissimilarity matrix. A constrained ordination (dbRDA) of the song‐type dissimilarity matrix overlaid with the most predictive acoustic characteristics (Figure 7) showed a similar pattern as the unconstrained NMDS of song types (Figure 4) and PCA of acoustic variables (Figure 6). Taken together, these analyses indicate that differences in song complexity (e.g., fewer frequency changes, shorter songs, fewer notes per syllable) differentiate song types of most individuals within the Puna region from populations at Keauhou Ranch, Hakalau National Wildlife Refuge, and the kīpuka region, but not individuals at Hawai'i Volcanoes National Park.

### 3.4 Ecological factors influencing acoustic characteristics

Examining ecological features that may be associated with differences in acoustic characteristics among populations, we found a significant positive linear correlation between acoustic characteristics and elevation while controlling for geographic distance between sites (*p* = .02, *r*_*s*_ = 0.22), which is consistent with the finding that low elevation sites in the Puna region are significantly different in acoustic characteristics compared to mid (e.g., kīpuka region) and high (e.g., Keauhou Ranch, Hakalau Forest National Wildlife Refuge) elevation sites in east Hawai'i. However, we did not find a relationship between acoustic characteristics and geographic distance when controlling for elevation (*p* = .64, *r*_*s*_ = −0.04). While the vertical structure of habitat influenced the acoustic characteristics of ‘amakihi songs (permanova; *F*_1,80_ = 4.60, *p* = .0006), based on an acoustic distance matrix of four principal component axes (Table S2), the influence was minimal (*R* ^2 ^= .05), especially when compared to population‐level differences in acoustic characteristics (Figure 6). Differences in acoustic characteristics between open and closed forest habitats were driven by principal component axis 3 which was maximally loaded by peak frequency (Tables S2 and S3). Songs produced by ‘amakihi from open forest habitats had more energy at higher frequencies ($\overset{¯}{x}$ peak frequency = 4760.78 ± 181.24 Hz) compared to songs from ‘amakihi in closed forest habitats ($\overset{¯}{x}$ peak frequency = 4309.03 ± 142.00 Hz). Assessment of the classification of habitat as open or closed forest using the point intercept method confirmed that the vertical structure of vegetation differed between the two forest habitat classifications (X^2 ^= 47.16, *df* = 7, *p* < .001) (Figure S2).

## 4 DISCUSSION

We found significant differences in both population song‐type repertoires and physical acoustic characteristics of ‘amakihi songs most strongly associated with the isolation of populations across an elevational gradient of disease susceptibility, and not the geographic distance among populations. Differences in ‘amakihi song were primarily driven by less complex songs (e.g., fewer frequency changes, shorter songs, fewer notes per syllable) of individuals recorded at low elevation sites in the Puna region compared to other populations at mid and high elevations. Structural differences in habitat played only a minimal role in acoustic differences among sites, suggesting that divergence in ‘amakihi song among populations is most likely attributed to the combined effects of population isolation across elevations, localized song learning and sharing, and cultural drift.

The unique history of low elevation ‘amakihi populations, which has recently emerged from a population bottleneck as a result of strong selection pressures of avian malaria and historical clearing of forest habitats, most likely contributed to the divergence of song‐type repertories of the Puna region from other populations at mid and high elevations. Avian malaria, an introduced disease that has negatively impacted population dynamics in the majority of Hawaiian honeycreepers (van Riper & Scott, 2001), restricts the current distribution of most Hawaiian honeycreepers to cool, high elevation forests where there is low prevalence of avian malaria (LaPointe et al., 2010; van Riper et al. 1987). However, rapidly expanding ‘amakihi populations have been documented in recent decades in low elevation areas such as the Puna region (Woodworth et al., 2005) with experimental studies indicating that low elevation populations have developed tolerance to avian malaria (Atkinson, Saili, Utzurrum, & Jarvi, 2013). Distinct genetic structuring between low elevation ‘amakihi in the Puna region and higher elevation populations that are still susceptible to avian malaria indicates that Puna region ‘amakihi expanded from remnant low elevation populations and were not recolonized from higher elevation populations (Eggert et al., 2008; Foster et al., 2007). The high mortality associated with avian malaria (Atkinson & LaPointe, 2009a,b) coupled with historical clearing of lowland forests (Pratt & Jacobi, 2009) most likely resulted in small, isolated populations of ‘amakihi that went undetected. Recent interbreeding between multiple remnant populations would explain the relatively high genetic diversity seen in present‐day low elevation ‘amakihi populations (Eggert et al., 2008; Foster et al., 2007). The combination of isolation and small population sizes of remnant low elevation populations may have led to the distinct song‐type repertoires and reduced song complexity that we observed in the present‐day Puna population through the influences of founder effects and cultural drift in song memes through time (Lynch, 1996).

Population size, along with isolation, has been shown to have important implications on the structure and diversity of song for a number of species following novel colonization events or a contraction in population size. For example, small numbers of North Island saddleback (*Philesturnus rufusater*) and North Island kōkako (*Callaeas wilsoni*) translocated to novel locations in New Zealand had lower acoustic variability and reduced song repertoire size compared to source populations, demonstrating the significant role of founder effects and cultural drift on song evolution in small, isolated populations (Parker et al., 2012; Valderrama, Molles, & Waas, 2013). Moreover, within isolated source populations as well as translocated populations of kōkako, population size was negatively associated with song sharing, such that smaller populations had higher levels of song sharing (e.g., lower song variability), most likely due to the lower availability and generation of novel song types (e.g., cultural mutations) from which young birds could learn (Valderrama, Molles, Waas, & Slabbekoorn, 2013). Similarly, studies of Dupont's lark (*Cherosphilus duponti*) within a fragmented landscape in Spain found increased song sharing and smaller population song repertoire sizes within smaller and more isolated habitat fragments that had fewer individuals (Laiolo & Tella, 2005, 2007). Taken together, these studies shed light on the potential evolutionary processes underlying the reduced acoustic variability and song repertoire complexity among ‘amakihi in the Puna region. While we cannot rule out that some variability in song structure across elevations may have already been present prior to the isolation of low elevation populations, the reduced complexity of song in the Puna region is consistent with the idea that avian malaria and habitat fragmentation, through their influence on population demographics, played an indirect role in shaping the acoustic characteristics of ‘amakihi populations at low elevations.

While there was considerable overlap in song‐type repertoires among ‘amakihi populations at mid and high elevations, individuals were more likely to share a song type with conspecifics within their population than from another population, suggesting some resistance to meme flow across the landscape. Historically, populations of ‘amakihi on Hawai'i Island inhabited more contiguous forest habitats across the island (Pratt & Jacobi, 2009). Large‐scale anthropogenic habitat destruction, introduced diseases, as well as natural lava flows have created isolating barriers that currently reduce the mixing of individuals between populations (Eggert et al., 2008), and potentially song sharing and meme flow across the landscape. We found evidence for reduced meme flow at a local scale (e.g., high variability in song‐type repertoires among sites) within the two populations that have the highest degree of fragmentation, the kīpuka and Hawai'i Volcanoes National Park. Sites within both locations consist of forest patches isolated from one another by relatively recent lava flows (e.g., 150 years old) that contain bare lava rock with scattered, small ‘ōhi'a trees and native shrubs creating a naturally fragmented landscape. Despite documented movement of ‘amakihi between forest patches in the kīpuka system (Knowlton et al., 2017), we still found significant variability in song‐type repertoires among patches which are spread across a relatively small area (~5 km), suggesting that structuring in ‘amakihi songs is due to more than just geographic isolation of populations.

We found little support for differences in acoustic characteristics of ‘amakihi songs associated with the vertical structure of forested habitats occupied by ‘amakihi, especially when compared to overall differences in acoustic characteristics among populations. Only peak frequency of an ‘amakihi's song differed between open and closed forest habitats. Our findings are consistent with a meta‐analysis examining evidence for the acoustic adaptation hypothesis that demonstrated adjusting peak frequency is a more efficient way to maximize broadcast efficacy of an individual's song compared to adjusting overall song frequency (Boncoraglio & Saino, 2007). While peak frequency differed between open and closed forest habitats, peak frequency was only a weak contributor to the variation we observed in acoustic characteristics across all study sites, suggesting that observed differences among populations are not likely to be due to structural differences in vegetation among study sites.

In conclusion, we found that ‘amakihi song varied most strongly across an elevational gradient that parallels disease susceptibility on the windward side of Hawai'i Island (LaPointe et al., 2010). The reduced complexity and variability of ‘amakihi songs in low elevation populations is most likely shaped by the effects of fragmentation and a disease‐driven population bottleneck, and maintained through isolation, localized song learning and sharing, and cultural drift. The current isolation and fragmentation of Hawaiian honeycreepers across the landscape is most strongly shaped by anthropogenic changes to forest habitats (e.g., urbanization, agricultural conversion, introduced plants and animals) and the distribution of *Culex quinquefasciatus*, a non‐native mosquito vector that transmits both avian malaria and avian pox (van Riper & Scott, 2001). However, both introduced pathogens and anthropogenic changes to the Hawaiian landscape are relatively recent in the evolutionary history of Hawaiian forest birds (Hawaiian honeycreeper lineage ~5 million years old; Lerner et al., 2011), and thus, divergence of ‘amakihi song as a result of isolation and fragmentation of populations may only be at the beginning stages. Continued isolation and fragmentation of forested habitats, as predicted by climate change models (Fortini, Vorsino, Amidon, Paxton, & Jacobi, 2015), may further intensify the divergence of ‘amakihi song, potentially inhibiting effective recognition and mixing of populations. In addition, non‐native diseases such as avian malaria likely inhibit meme flow across elevations given the low survival of high elevation susceptible individuals at low elevations, and the potential for outbreeding depression of individuals adapted to high disease pressure moving to higher elevations where malaria is not present. These results indicate the dynamic nature of a species’ acoustic characteristics, which are sensitive to landscape and demographic forces. As many species around the world face large changes to their habitat and population dynamics, understanding the current diversity of song across the geographic range of species not only preserves the cultural heritage of a species, but also provides insight into how species are adapting to change.

## AUTHOR CONTRIBUTION

JPC, PJH, EHP, and AAP conceived the idea, design, experiment; JPC conducted the research; JPC and KLP analyzed the data and wrote the manuscript; PJH, EHP, and AAP made substantial edits to the manuscript.
