# Supplementary material for: The effect of isolation, fragmentation, and population bottlenecks on song structure of a Hawaiian honeycreeper
Source: Ecol Evol. 2018 Jan 18;8(4):2076–87. doi: 10.1002/ece3.3820 (PMC5817154; doi:10.1002/ece3.3820)
Supplement: Supplementary file 1 [file ECE3-8-2076-s001.pdf]

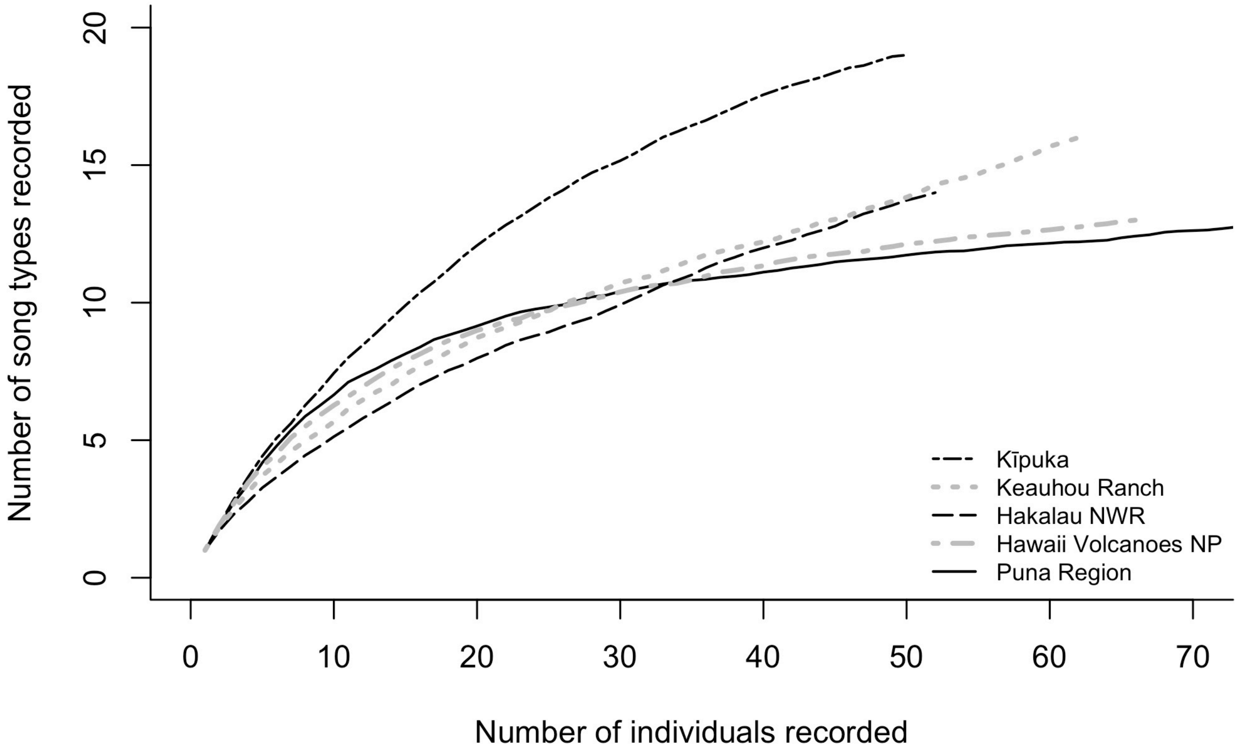

Figure S1. Accumulation curve of the number of song types recorded at each location relative to the number of individuals recorded. The number of individuals recorded is truncated at 70 individuals; however, the pattern doesn't change with increased sample sizes for the Puna region, the only site with greater than 70 individuals recorded.
