# Supplementary material for: The effect of isolation, fragmentation, and population bottlenecks on song structure of a Hawaiian honeycreeper
Source: Ecol Evol. 2018 Jan 18;8(4):2076–87. doi: 10.1002/ece3.3820 (PMC5817154; doi:10.1002/ece3.3820)
Supplement: Supplementary file 2 [file ECE3-8-2076-s002.pdf]

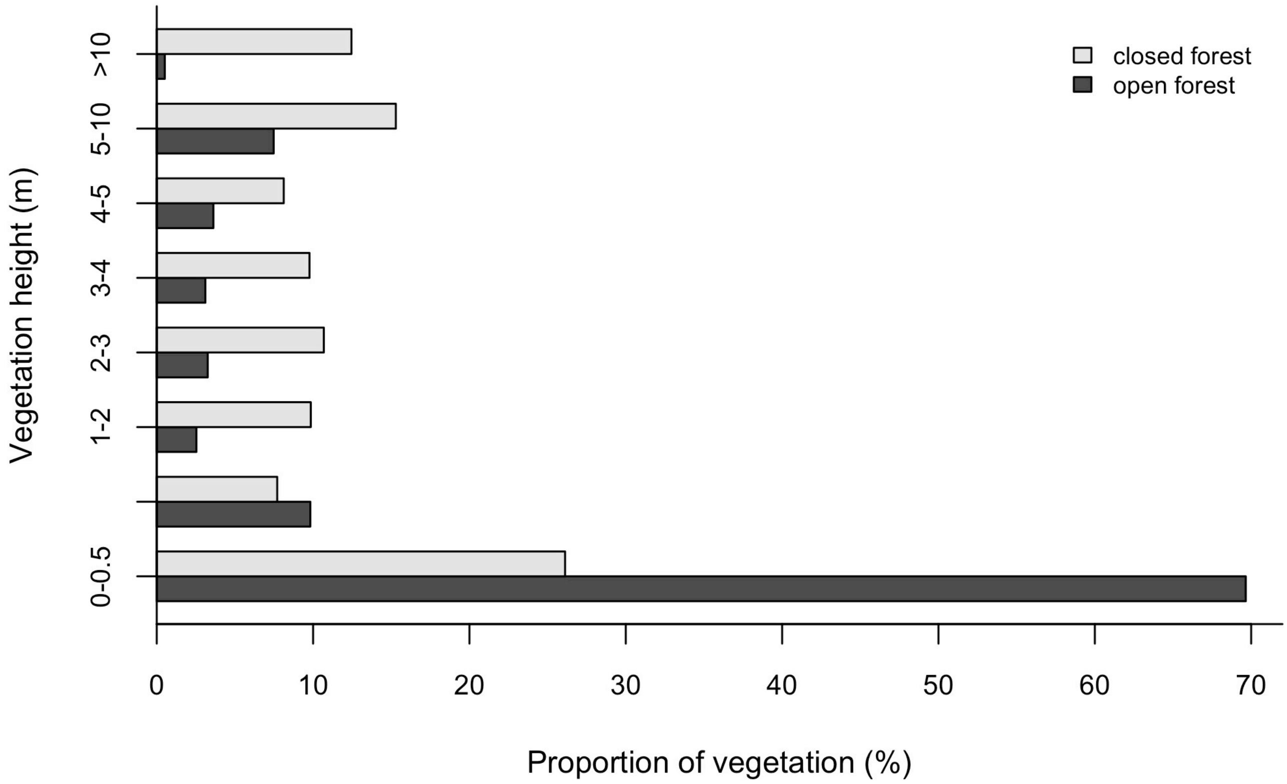

Figure S2. Vertical vegetation profile of habitats classified as open and closed forests at Hakalau National Wildlife Refuge and Keauhou Ranch. The distribution of vegetation within closed forest habitats was more evenly distributed across the vertical vegetation profile compared to open forest habitats, which had a dense ground cover of non-native grasses with little mid-story structuring, and a semi-open canopy. Unlike open forest habitats, ground cover of closed forest habitats was comprised of native ferns, tree and shrub saplings, and interspersed native and introduced grasses.
