# Supplementary material for: The effect of isolation, fragmentation, and population bottlenecks on song structure of a Hawaiian honeycreeper
Source: Ecol Evol. 2018 Jan 18;8(4):2076–87. doi: 10.1002/ece3.3820 (PMC5817154; doi:10.1002/ece3.3820)
Supplement: Supplementary file 3 [file ECE3-8-2076-s003.pdf]

Table S1. Post-hoc pair-wise comparisons of each site to test for differences in: 1) song type and 2) acoustic characteristics among locations (e.g., PERMANOVA), and 3) variability of song types within locations (e.g., PERMDISP).

	PERMANOVA		PERMDISP
Comparison	Song Type	Acoustic Characteristics	Song Type
HAVO vs. HNWR	0.20	0.29	0.09
HAVO vs. KEAU	0.14	0.21	**0.03**
HAVO vs. KIPU	0.76	**0.03**	0.82
HAVO vs. PUNA	0.56	**0.004**	**0.05**
HNWR vs. KEAU	0.20	0.7	0.85
HNWR vs. KIPU	0.06	0.14	**0.05**
HNWR vs. PUNA	**0.03**	**0.01**	0.58
KEAU vs. KIPU	0.30	**0.03**	**0.02**
KEAU vs. PUNA	**0.01**	**0.009**	0.68
KIPU vs. PUNA	**0.01**	**0.004**	**0.02**
