# Supplementary material for: The effect of isolation, fragmentation, and population bottlenecks on song structure of a Hawaiian honeycreeper
Source: Ecol Evol. 2018 Jan 18;8(4):2076–87. doi: 10.1002/ece3.3820 (PMC5817154; doi:10.1002/ece3.3820)
Supplement: Supplementary file 4 [file ECE3-8-2076-s004.pdf]

Table S2. Principal component loading values of eight acoustic variables measured for Hawaiʻi ʻamakihi songs recorded within open and closed understory forested habitat at Keauhou Ranch and Hakalau National Wildlife Refuge. Four principal component axes had eigenvalues greater than 1, explaining 80% of the variation in acoustic variables.

Acoustic variables	PC1	PC2	PC3	PC4
song length	-0.31	0.62		0.10
low freq	0.50	0.23		0.41
high freq	-0.36	-0.24	-0.28	0.60
freq bandwidth	-0.60	-0.32		
peak freq	-0.18		0.53	-0.37
freq change	-0.12		-0.53	-0.52
ln(no. syllables)	-0.34	0.54	0.28	0.14
ln(notes per syllable)		0.33	-0.51	-0.18
Eigenvalue	2.22	1.73	1.33	1.03
Proportion of variation	0.28	0.22	0.17	0.13
Cumulative proportion	0.28	0.50	0.67	0.80
