# Supplementary material for: The effect of isolation, fragmentation, and population bottlenecks on song structure of a Hawaiian honeycreeper
Source: Ecol Evol. 2018 Jan 18;8(4):2076–87. doi: 10.1002/ece3.3820 (PMC5817154; doi:10.1002/ece3.3820)
Supplement: Supplementary file 5 [file ECE3-8-2076-s005.pdf]

Table S3. Mean and 95% confidence intervals (CI) of the eight acoustic characteristics measured from Hawai'i 'amakihi songs recorded within open and closed understory forested habitat at Keauhou Ranch and Hakalau National Wildlife Refuge. In addition, the mean and 95% CI is shown for the four principal component (PC) axes that explained the most variability in acoustic variables. The only acoustic variable with non-overlapping 95% CI was peak frequency which loaded strongest on PC3, the only PC axis with non-overlapping 95% CI. Variables with non-overlapping 95% CI values are shown in bold.

		95% CI			95% CI	
Acoustic variables	Open forest	Lower	Upper	Closed forest	Lower	Upper
song length	1.73	1.66	1.81	1.79	1.73	1.85
low freq	1815.45	1734.61	1896.29	1894.46	1831.94	1956.97
high freq	5902.61	5836.38	5968.84	5934.63	5884.56	5984.69
freq bandwidth	4087.17	3975.32	4199.03	4040.17	3962.11	4118.22
**peak freq**	**4760.78**	**4579.54**	**4942.02**	**4309.03**	**4167.03**	**4451.03**
freq change	3.14	2.86	3.41	3.30	3.07	3.53
ln(no. syllables)	2.19	2.14	2.24	2.12	2.07	2.16
ln(notes per syllable)	0.59	0.47	0.73	0.86	0.73	0.98
PC1	-0.20	-0.74	0.35	0.18	-0.19	0.55
PC2	-0.13	-0.58	0.33	0.11	-0.25	0.48
**PC3**	**0.55**	**0.22**	**0.88**	**-0.50**	**-0.81**	**-0.19**
PC4	-0.14	-0.52	0.24	0.13	-0.11	0.37
